# Relapse or re-infection after BPaL treatment for pre-extensively drug-resistant TB

**DOI:** 10.5588/ijtldopen.26.0488

**Published:** 2026-11-13

**Authors:** T.T. Thuy Hoang, T.U. Ca Dang, T.H. Nguyen, A. Gebhard, S. Foraida, M. Diachenko

**Affiliations:** 1 National TB Control Programme of Vietnam, National Lung Hospital, Hanoi, Vietnam;; 2 Pham Ngoc Thach Hospital, Ho Chi Minh City, Vietnam;; 3 KNCV Tuberculosis Foundation, Hanoi, Vietnam;; 4 KNCV Tuberculosis Foundation, The Hague, the Netherlands;; 5 TB Alliance, New York, NY, USA.

**Keywords:** tuberculosis, rifampicin-resistant TB, post-treatment surveillance, sustained treatment success, linezolid toxicity

Dear Editor,

The BPaL regimen (bedaquiline, pretomanid, and linezolid) has improved the treatment of multidrug-resistant TB and pre-extensively drug-resistant TB (pre-XDR-TB) by providing an all-oral, shorter-course regimen with high treatment success rates.^1,2^ However, evidence regarding the durability of treatment outcomes and post-treatment recurrence remains limited.^3^ The WHO has emphasised the importance of sustained treatment success, defined as being alive and free of TB after successful treatment completion.^4^

We report a case of recurrent rifampicin-resistant TB (RR-TB) diagnosed 18 months after successful BPaL treatment for pre-XDR-TB. Although relapse and re-infection could not be distinguished, the case highlights the need for continued post-treatment surveillance, careful patient history taking for any previous treatments and DST to establish drug-resistance patterns as well as the valuable role of genetic typing.

A 56-year-old HIV-negative man with no significant comorbidities presented with chronic productive cough and constitutional symptoms in February 2022. He sought care at a provincial TB hospital because of persistent respiratory symptoms and worsening fatigue. Sputum smear microscopy was positive (AFB 2+), MGIT culture confirmed *Mycobacterium tuberculosis*, and Xpert MTB/RIF detected rifampicin resistance. A second-line line probe assay demonstrated fluoroquinolone resistance, leading to a diagnosis of pre-XDR-TB. The patient initiated a 6-month BPaL regimen in April 2022, consisting of bedaquiline, pretomanid, and linezolid (1,200 mg). Bedaquiline was administered daily for the first 2 weeks, followed by three times weekly dosing. All other drugs were administered daily. The patient was hospitalised during the first month for close monitoring of adverse events, after which treatment continued on an outpatient basis with weekly drug dispensing, remote adherence monitoring, and monthly clinical follow-up visits. Ethical statement: the patient's treatment regimen was reviewed and approved by the National Tuberculosis Expert Clinical Committee prior to treatment initiation. Written informed consent for treatment was obtained from the patient. All potentially identifiable information has been omitted to protect patient confidentiality.

Baseline haematological parameters were within the laboratory reference range (red blood cell [RBC] 4.46 × 10^12^/L; haemoglobin 14.3 g/dL). During treatment, grade 2 anaemia developed at week 10 (RBC 3.01 × 10^12^/L; haemoglobin 9.6 g/dL). Despite supportive management, anaemia progressed to grade 4 by week 13 (RBC 1.81 × 10^12^/L; haemoglobin 5.7 g/dL). Linezolid was temporarily discontinued (Figure), while bedaquiline and pretomanid were continued, and the patient received blood transfusion. Following haematological recovery at week 15 (RBC 2.84 × 10^12^/L; haemoglobin 8.7 g/dL), linezolid was reintroduced at a reduced dose of 300 mg daily with close monitoring. The patient completed the planned treatment course without documented treatment interruption other than the temporary linezolid discontinuation and consequent linezolid dose reduction, achieving sputum smear and culture conversion, with stable body weight during follow-up (Table), and successfully completed therapy in October 2022.

**Figure.**
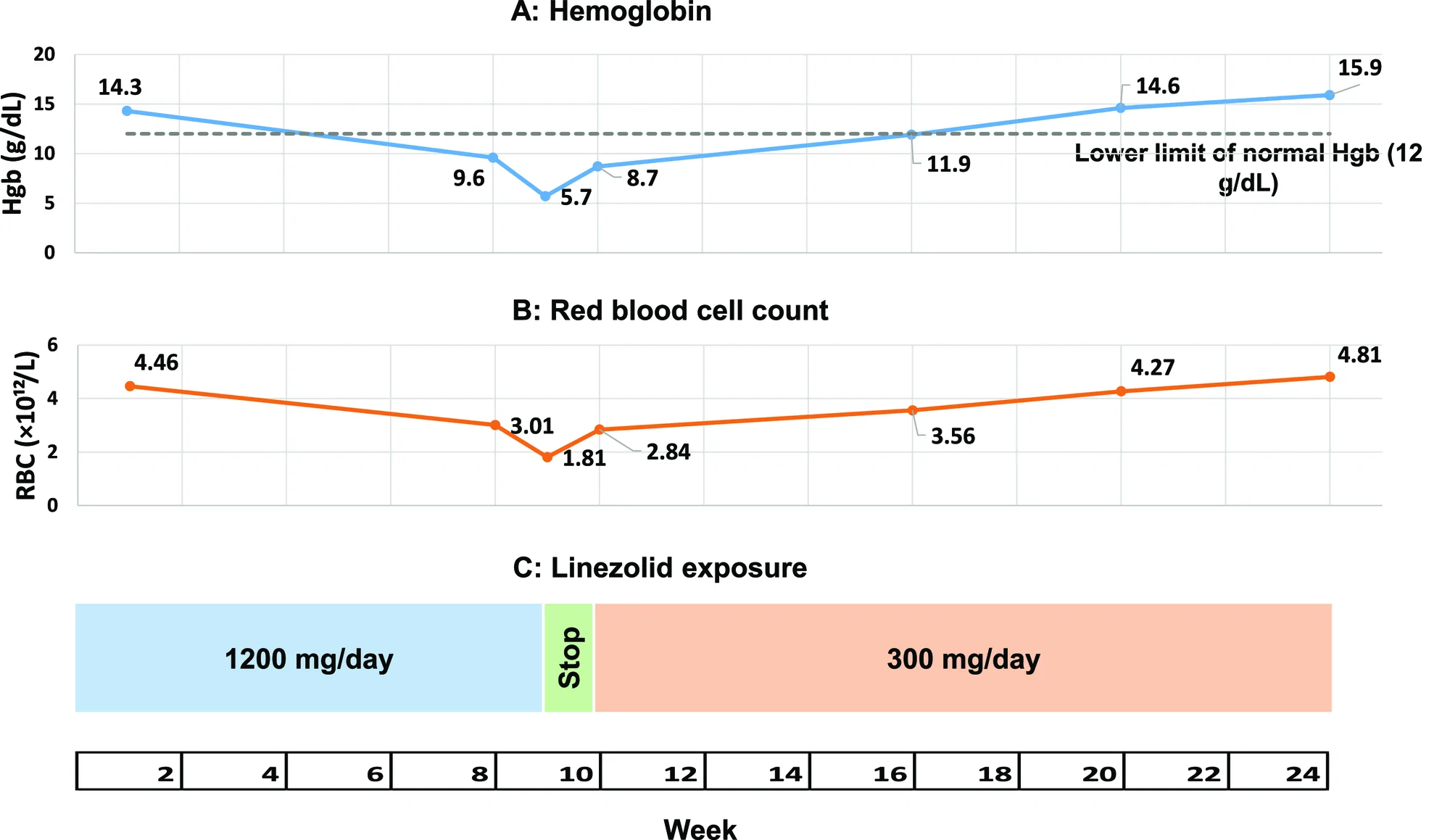
Haematologic changes and linezolid dose modifications during treatment. **A:** Haemoglobin levels over time; **B:** red blood cell (RBC) count over time; **C:** timeline of linezolid dosing, including discontinuation at week 9 and reintroduction at a reduced dose of 300 mg/day from week 10.

**Table.** Baseline bacteriological findings and treatment response during BPaL therapy.

Parameter	Baseline	Month 1	Month 2	Month 3	Month 4	Month 5	Month 6
Smear	AFB 2+	Neg	Neg	Neg	NA	Neg	Neg
Culture	Positive	Positive	Neg	Neg	NA	Neg	Neg
Weight (kg)	65.0	65.0	65.0	65.5	65.5	66.0	66.0

AFB = acid-fast bacilli; NA = not available; Neg = negative.

In 2024, 18 months after treatment completion, the patient was diagnosed with recurrent RR-TB based on clinical, bacteriological, and drug susceptibility testing. MGIT culture was positive for *M. tuberculosis*, and first-line drug susceptibility testing showed resistance to rifampicin, streptomycin, isoniazid, and ethambutol. However, second-line line probe assay demonstrated susceptibility to fluoroquinolones, contrasting with the fluoroquinolone-resistant result before BPaL treatment. The patient lived with his wife, who had been diagnosed with RR-TB in February 2022. Unlike the patient, her baseline drug susceptibility testing showed fluoroquinolone susceptibility. She achieved sputum culture conversion after 2 months of treatment and completed an 18-month treatment regimen successfully in September 2023.

This case highlights potential challenges regarding the long-term effectiveness of the BPaL regimen for pre-XDR-TB. BPaL has demonstrated high treatment success rates in clinical trials and real-world settings.^2,5^ Pretomanid-based regimens have also been shown to be cost-effective in selected contexts.^6^ However, evidence regarding the durability of treatment outcomes and post-treatment recurrence remains limited.^2,3^ The recurrence observed 18 months after treatment completion illustrates that treatment success at the end of therapy may not necessarily equate to long-term TB-free survival. This observation is consistent with the WHO’s notion of sustained treatment success and the need to evaluate outcomes beyond treatment completion.^4^

The key unresolved question in this case is whether the recurrent episode represented relapse of the original infection or re-infection with a new strain. Definitive differentiation was not possible in the absence of molecular typing of isolates from the two episodes. Several findings may support relapse. Severe linezolid-associated haematological toxicity required dose reduction and temporary interruption of therapy. As linezolid is a key component of the BPaL regimen, contributing to its bactericidal and sterilising activity,^2^ reduced drug exposure may have attenuated regimen activity. The initial use of high-dose linezolid (1,200 mg daily) may also have contributed to toxicity, and WHO guidance supports lower dosing (600 mg daily) to improve tolerability while maintaining efficacy.^2,4^ The change in the fluoroquinolone susceptibility pattern between episodes does not exclude relapse. One possible explanation is fluoroquinolone heteroresistance, defined as the coexistence of susceptible and resistant bacillary populations within the same specimen.^7^ Heteroresistance may arise from resistant and susceptible subpopulations within a single strain due to within-host microevolution,^8^ or from mixed infection with multiple *M. tuberculosis* strains.^9^ Treatment-related shifts in these populations may result in recurrence with an apparently different resistance profile. Another explanation relates to limitations of molecular drug susceptibility testing. Commercial line probe assays interrogate only selected resistance-associated regions and may fail to detect resistance when mutant bacilli are present at low frequency or when bacterial load is low, particularly in heteroresistant or post-treatment settings.^10^ Consequently, variation in the proportion of mutant bacilli between episodes may yield discrepant fluoroquinolone susceptibility results despite persistence of the same strain.

Conversely, several findings may suggest re-infection. The recurrent episode showed rifampicin resistance with no detectable fluoroquinolone resistance on line probe assay, which is compatible with infection by a different strain; however, this cannot be confirmed without molecular typing. In high-burden settings, whole-genome sequencing (WGS) studies have shown that re-infection contributes substantially to recurrent TB after treatment completion.^11^ However, this is unlikely to be attributable to exposure from the patient's wife, as she had already achieved sputum culture conversion before her husband completed his treatment. Community-acquired transmission therefore remains a plausible explanation, consistent with molecular epidemiological studies from Vietnam, suggesting that a substantial proportion of TB transmission occurs outside the household.^12^ Although re-infection may be considered plausible given the epidemiological context and changes in drug-resistance patterns, relapse cannot be excluded without molecular evidence. Wider use of WGS would help distinguish between these mechanisms and clarify the origin of recurrent disease.^13^

Regardless of the underlying mechanism, optimal management of drug-resistant TB should extend beyond selection of an effective regimen and include careful monitoring of adverse events, maintenance of treatment adherence, post-treatment follow-up, and strengthened infection control measures. Integration of molecular epidemiological tools, particularly WGS, may help distinguish relapse from re-infection and guide future prevention and management strategies. Relapse or re-infection may occur even after successful completion of BPaL treatment in patients with pre-XDR-TB, particularly when treatment-limiting toxicity and subsequent exposure to *M. tuberculosis* remain possible. End-of-treatment bacteriological conversion does not necessarily guarantee long-term TB-free survival, underscoring the need for long-term post-treatment follow-up and effective infection control. The case highlights the importance of careful assessment of TB treatment history, drug susceptibility testing for resistance patterns, and genetic typing to distinguish relapse from re-infection. Further studies are needed to evaluate the long-term durability of BPaL outcomes and identify predictors of recurrence.
